# On the weight of indels in genomic distances

**DOI:** 10.1186/1471-2105-12-S9-S13

**Published:** 2011-10-05

**Authors:** Marília D V Braga, Raphael Machado, Leonardo C Ribeiro, Jens Stoye

**Affiliations:** 1 Instituto Nacional de Metrologia, Qualidade e Tecnologia, Duque de Caxias, 25250-020, Brazil; 2 AG Genominformatik, Technische Fakultät, Universität Bielefeld, Bielefeld, 33594, Germany

## Abstract

**Background:**

Classical approaches to compute the genomic distance are usually limited to genomes with the same content, without duplicated markers. However, differences in the gene content are frequently observed and can reflect important evolutionary aspects. A few polynomial time algorithms that include genome rearrangements, insertions and deletions (or substitutions) were already proposed. These methods often allow a block of contiguous markers to be inserted, deleted or substituted at once but result in distance functions that do not respect the triangular inequality and hence do not constitute metrics.

**Results:**

In the present study we discuss the disruption of the triangular inequality in some of the available methods and give a framework to establish an efficient correction for two models recently proposed, one that includes insertions, deletions and double cut and join (DCJ) operations, and one that includes substitutions and DCJ operations.

**Conclusions:**

We show that the proposed framework establishes the triangular inequality in both distances, by summing a surcharge on indel operations and on substitutions that depends only on the number of markers affected by these operations. This correction can be applied *a posteriori*, without interfering with the already available formulas to compute these distances. We claim that this correction leads to distances that are biologically more plausible.

## Background

The approaches to compute the distance between two genomes often allow the rearrangement operations to be applied to blocks of contiguous markers of arbitrary sizes. In this context, the genomes are assumed to have the same content, free of duplicated markers, and the allowed operations only change the organization of the genome (i.e. positions and orientation of markers, number and type of chromosomes, etc.). We call these operations *organizational*. Furthermore, the classical polynomial time approaches usually assign the same weight to all organizational operations regardless of the size of the affected blocks and the particular types of the operations, that could represent inversions, translocations, fusions and fissions [1-4].

While duplicated markers can hardly be handled by exact models [5-8], some extensions of the classical approaches lead to *hybrid* models that handle genomes with unequal content, but without duplicated markers, allowing, in addition to the organizational operations, a block of contiguous markers to be inserted, deleted or substituted at once [9-12]. Insertions, deletions and substitutions are called *content-modifying* operations. The hybrid models that we analyze in the present study assign the same weight to organizational and content-modifying operations and lead to exact polynomial time algorithms. However, they compute distances that do not necessarily respect the triangular inequality. Although the triangular inequality disruption does not affect pairwise comparisons, this may be a major issue if one intends to use these genomic distances to compute the median of three or more genomes and in phylogenetic reconstructions.

By assigning different weights to different types of operations one can avoid the triangular inequality disruption. These weights should actually be guided by biological evolution criteria, but the lack of biological understanding makes this task still difficult. In the present work we investigate how the triangular inequality itself can be used to determine some constraints on the weights of rearrangement operations. Considering in particular two hybrid models recently proposed by us [10,11], in which double-cut-and-joins (DCJ) represent the organizational operations, we propose a general framework to establish the triangular inequality in these models, improving our previous results.

In the remainder of this section we will introduce some preliminary concepts and give an overview of two different hybrid models available in the literature, namely the *inversion-indel* distance [9] and the *ghost-DCJ* distance [12]. We will then summarize our *DCJ-indel*[10] and *DCJ-substitution* distances [11], that are the basis for the results obtained in the present work.

### Genomes

We deal with models in which duplicated markers are not allowed. Given two genomes *A* and *B*, possibly with unequal content, let  be three disjoint sets, such that  is the set of markers that occur once in *A* and once in *B*,  is the set of markers that occur only in *A* and  is the set of markers that occur only in *B.* The markers in sets  are also called *unique markers.* We denote by  the number of unique markers in genomes *A* and *B.*

Each marker *g* in a genome is a DNA fragment and is represented by the symbol *g*, if it is read in direct orientation, or by the symbol *ḡ*, if it is read in reverse orientation. Each one of the two extremities of a linear chromosome is called a *telomere*, represented by the symbol ○. Each chromosome in a genome can be then represented by a string that can be circular, if the chromosome is circular, or linear and flanked by the symbols ○ if the chromosome is linear.

### Organizational operations

The organizational operations change the organization of a genome, without changing its content [1]. Several types of organizational operations are considered and can be represented as follows. An *inversion* is an operation that reverses the order and the orientation of a block of contiguous markers. An inversion applied to markers *b*, c and *d* of the linear chromosome ○*abcde*○ results in the linear chromosome . By a *translocation*, a pair of linear chromosomes exchange blocks of contiguous markers located at one of the extremities. A translocation applied to ○*abcd*○ and ○*efg*○ could result in chromosomes ○*abfg*○ and ○*ecd*○, for example. Similarly, a *fusion* of a pair of chromosomes ○*abcd*○ and ○*efg*○ could result in ○*abcdefg*○, while the opposite operation is a *fission.*

All rearrangements listed above can be generically represented as a *double-cut-and-join* (DCJ), that is the operation that generally performs two cuts in a genome, creating four open ends, and joins these open ends in a different way. This operation was introduced in 2005 by Yancopoulos *et al.*[*2*].

### Content-modifying operations

The content-modifying operations change the content of a genome. These operations can be a *deletion* of a block of contiguous markers or an *insertion* of a block of contiguous markers, with the restriction that an insertion cannot produce duplicated markers. As an example, a deletion of markers *x*, *y* and *z* from a chromosome ○*abxyzc*○ results in ○*abc*○. The opposite of a deletion is an insertion. Insertions and deletions can be simply called *indel* operations.

We also consider a more parsimonious operation, in which a block of contiguous markers can be substituted by a different block of contiguous markers, also with the restriction that a *substitution* cannot produce duplicated markers. An example of a substitution could transform o*abxyzc*o into o*abuvc*o. The opposite of a substitution is also a substitution. Furthermore, each one of the considered blocks can be empty, allowing a substitution to represent an insertion or a deletion. At most one chromosome can be inserted, deleted or substituted at once.

### Triangular inequality

Given any three genomes *A*, *B* and *C* and a distance measure *d*, consider without loss of generality that *d*(*A*, *B*) ≥ *d*(*A*, *C*) and *d*(*A*, *B*) ≥ *d*(*B*, *C*). Then the triangular inequality is the property that guarantees that *d*(*A*, *B*) ≤ *d*(*A*, *C*) + *d*(*B*, *C*). Although this property holds for the classical models that consider only organizational operations, it does not hold for the hybrid approaches that we analyze in this study.

Consider for example the genomes [12]. While *A* and *B* can be sorted into *C* with only one indel, the minimum number of inversions required to sort *A* into *B* is three. In this case we have *d*(*A*, *B*) = 3, *d*(*A*, *C*) = 1, *d*(*B*, *C*) = 1 and the triangular inequality is disrupted. The triangular inequality disruption may be a serious obstacle if one intends to use the distance to compute the median of three or more genomes and in phylogenetic reconstructions.

### Related work

#### The inversion-indel distance

El Mabrouk [9] extended the classical sorting by inversions approach [1] to develop a method to compare unichromosomal genomes considering inversions and indels. Two algorithms were provided, an exact one, which deals with insertions and deletions asymmetrically, and a heuristic that is able to handle all operations symmetrically. The triangular inequality can be disrupted in this model, as we could see in the example above, but this issue was not discussed by the author.

#### The ghost-DCJ distance

Yancopoulos and Friedberg [12] proposed an extension of the classical DCJ model [2], leading to a hybrid model that considers DCJ operations and indels. In their approach, they give a method to insert ghost markers in the genomes, so that the contents are equalized and can be sorted with DCJ operations only. With such a strategy, indels are mimicked by DCJ operations, and it is actually not possible to make a clear separation between organizational and content-modifying operations.

The triangular inequality disruption was detected by the authors and an approach to avoid this problem was proposed, imposing a kind of constraint to the ghost insertion. However, in comparisons involving three genomes, by the insertion of ghosts a genome could be modified in different ways, depending on the second genome. Consider again the genomes . It is necessary to insert ghosts in *C*, generating a modified genome *C*′, so that *d*(*A*, *C*′) is minimized. We have  and *d*(*A*, *C*′) = 2, but in this process genome *B* was not considered. In the same way, while inserting ghosts in *C* with respect to *B* to generate a modified genome *C*″, genome *A* is not considered. We have *C*″ = *C* ∪ {*bcd*} and *d*(*A*, *C*″) = 2. Since we have *d*(*A*, *B*) = 3, the triangular inequality holds. But the genomes *C*′ and *C*″ are actually different and there is no analysis of the impact of these differences. In this case, for instance, we have *d*(*A*, *C*″) = *d*(*B*, *C*′) = 3. Moreover, the genomes *C*′ and *C*″ are composed of one linear and one circular chromosome. We observe that in general, the insertion of ghosts leads to the insertion of one or more circular chromosomes in the modified genomes, regardless of the fact that the original genome is linear.

### The DCJ-indel and DCJ-substitution distances

The basis for the results of the present work are two hybrid models recently developed by us [10,11], by doing a different extension of the classical DCJ model [2,3]. In [10] the considered operations are DCJs and indels, while in [11] we consider DCJs and substitutions (that comprehend indels). Differently from the approach of Yancopoulos *et al.*[*12*], a clear separation between organizational and content-modifying operations is provided. The *DCJ-indel distance* of *A* and *B*, denoted by *d^DCJ^*^–^*^id^*(*A*, *B*), is the minimum number of DCJs and indels required to transform *A* into *B.* Similarly, the *DCJ-substitution distance* of *A* and *B*, denoted by *d^DCJ^*^–^*^sb^*(*A*, *B*), is the minimum number of DCJs and substitutions required to transform *A* into *B.* Since substitutions include indels, *d^DCJ^*^–^*^sb^*(*A*,*B*) is upper bounded by *d^DCJ^*^–^*^id^*(*A*,*B*). Both distances can be computed in linear time, but are subject to the inequality disruption. We give some details of the algorithms to compute both distances in the following.

#### The classical DCJ distance

Given two genomes *A* and *B*, recall that  is the set of markers common to *A* and *B.* The two extremities of each marker , are denoted *g^t^* (tail) and *g^h^* (head). A [10] in genome *A* (respectively in genome *B*) is in general a linear string *v* = *γ*_1_ℓ*γ*_2_, such that each *γ_i_* can be a telomere or an extremity of a marker from . The string ℓ is the *label* of *v:* it is composed of the markers that are between *γ*_1_ and *γ*_2_ in *A* (respectively in *B*) and contains no marker that also belongs to . If a linear chromosome is composed only of markers that are not in , it is represented by a  ○ℓ○. Similarly if a circular chromosome is composed only of markers that are not in , it is represented by a ℓ. In this particular case we have a circular instead of a linear string representing an adjacency.

**Figure 1:**
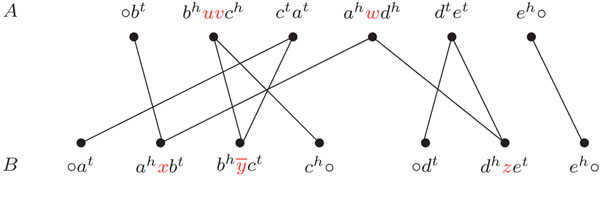
For genomes *A* and *B*, the adjacency graph contains one *BB*-path and two *AB*-paths.

Each  in genome *A* and each  in genome *B* corresponds to a vertex in the *adjacency graph AG*(*A*, *B*) [*3*]. For each , we have one edge connecting the vertex in *A* and the vertex in *B* that contain *g^h^* and one edge connecting the vertex in *A* and the vertex in *B* that contain *g^t^.* The graph *AG*(*A*, *B*) is bipartite, composed of connected components that alternate vertices in genome *A* and in genome *B.* Each component can be either a cycle, or an *AB-path* (that have one endpoint in genome *A* and the other in B), or an *AA-path* (that have both endpoints in genome *A*), or a *BB-path* (that have both endpoints in B). A component can also be a *linear* (respect. *circular*) *singleton*, that is a linear (respect. circular) chromosome represented by a single . The number of vertices in a component *P* of *AG*(*A*, *B*) is denoted by |*P*|. An example of an adjacency graph is given in Figure 1.

Components with 3 or more vertices need to be reduced, by applying DCJ operations, to components with only 2 vertices, that can be cycles or *AB*-paths. This procedure is called *DCJ-sorting* of *A* into *B.* The number of *AB*-paths in *AG*(*A*, *B*) is always even and a DCJ operation can be of three types [3]: it can either decrease the number of cycles by one, or the number of *AB*-paths by two; or it does not affect the number of cycles and *AB*-paths; or it can either increase the number of cycles by one, or the number of *AB*-paths by two. In the last case the DCJ operation is called *optimal.* It is possible to do a DCJ-sorting with optimal DCJs only [3]. The minimum number of steps required to do a DCJ-sorting of *A* into B is the *DCJ distance* of *A* and B, denoted by *d^DCJ^*(*A*, *B*), that can be then computed by the following formula:

**Theorem 1** ( [3]) *Given two genomes A and B without duplicated markers*, *we have*, *where n is the number of common markers between A and B*, *and c and b are the number of cycles and of AB-paths in AG*(*A*,*B*), *respectively.*

#### Runs of unique markers and tight distance upper bounds

**Figure 2:**
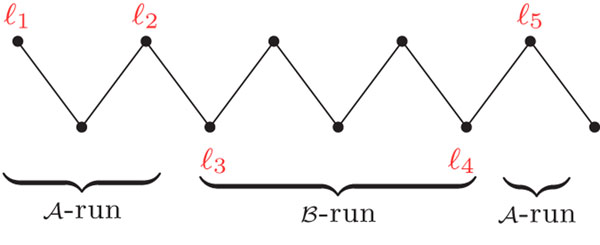
An *AB*-path with 3 runs.

We can obtain a string ℓ(*P*) by concatenating only the labels of the vertices of a component *P* of *AG*(*A*, *B*). We have to be careful if *P* is a cycle and has labels in both genomes *A* and B. In this case we need to start to read between a labeled  of *A* and a labeled  of *B*; otherwise *P* has labels in at most one genome and we can start anywhere. An  (respectively a ) is then a maximal substring of ℓ(*P*) composed only of markers in (respectively in ). Each  or  can be simply called *run.* We denote by Λ(*P*) the number of runs in a component *P* (see an example in Figure 2). Observe that Λ(*P*) ≤ |*P*|, where |*P*| is the number of vertices in component *P.*

A set of labels of one genome can be *accumulated* with DCJs. In particular, when we apply optimal DCJs on only one component of the adjacency graph, we can accumulate an entire run in a single [10]. Runs can also be merged by DCJ operations. Consequently, while sorting a genome into another with DCJs, we can reduce the overall number of runs. In the end of this process, each run can be sorted with one indel operation. Alternatively, a pair of consecutive runs can be sorted with one substitution.

It is possible to do a separate DCJ-sorting using only optimal DCJs in any component *P* of *AG*(*A*, *B*) [4]. We denote by *d^DCJ^* (*P*) the number of DCJ operations used for DCJ-sorting *P* separately. The DCJ distance can also be re-written as *d^DCJ^*(*A*, *B*) = ∑*_P_*_∈_*_AG_*_(_*_A_*_,_*_B_*_)_*d^DCJ^*(*P*) [4]. Then, the *indel-potential* of *P*, denoted by *λ*(*P*), is defined as the minimum number of runs that we can obtain by doing a separate DCJ-sorting in *P* with *d^DCJ^* (*P*) DCJ operations. It can be computed with a simple formula that depends only on the number of runs in P: , if Λ(*P*) ≥ 1 (otherwise *λ*(*P*) = 0) [10]. This gives a tight upper bound for the DCJ-indel distance:

**Lemma 1** ( [10]) *Given two genomes A and B without duplicated markers*, *we have*

Similarly, the *substitution-potential* of a component P, that is the minimum number of substitutions that we can obtain by DCJ-sorting *P* with *d^DCJ^* (*P*) DCJ operations, is denoted by *σ*(*P*) and can be computed as follows: , if Λ(*P*) ≥ 1 (otherwise *σ*(*P*) = 0) [11]. With the substitution-potential we also have a tight upper bound for the DCJ-substitution distance:

**Lemma 2** ( [11]) *Given two genomes A and B without duplicated markers*, *we have*

Based on the upper bounds above and some additional technical aspects that we omit here, it is possible to exactly compute both distances in linear time [10,11].

#### Establishing the triangular inequality

**Figure 3:**
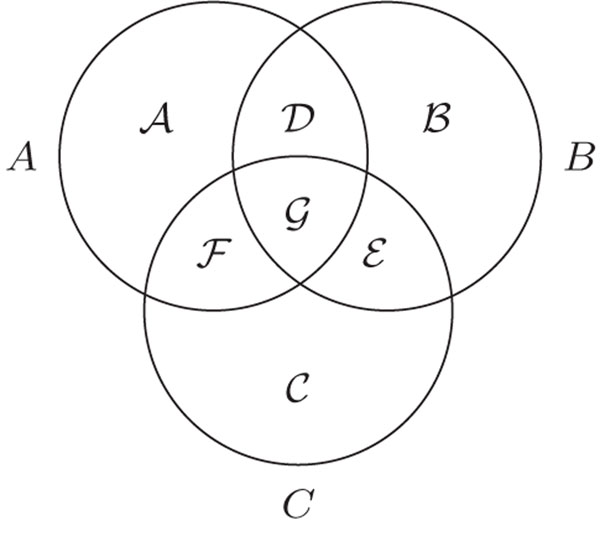
The disjoint sets  for three genomes *A*, *B* and *C* - each circle represents the markers that occur in each one of the three genomes.

In the case of the DCJ-indel distance, there is a method to establish the triangular inequality *a posteriori*[*10*]. Let *A*, *B* and *C* be three genomes and let  be seven disjoint sets of markers, such that  are the sets of unique markers that occur respectively only in *A*, *B* and *C.* Furthermore, the markers in  are common only to *A* and *B*, the markers in  are common only to *B* and *C*, the markers in  are common only to *A* and *C*, and, finally,  is the set of markers that are common to *A*, *B* and *C.* The sets  are represented in Figure 3.

Consider without loss of generality that *d^DCJ^*^–^*^id^*(*A*, *B*) ≥ *d^DCJ^*^–^*^id^*(*A*, *C*) and *d^DCJ^*^–^*^id^*(*A*, *B*) ≥ *d^DCJ^*^–^*^id^*(*B*, *C*). If , meaning that genomes *A* and *B* have no common marker that does not occur in *C*, the triangular inequality holds for the DCJ-indel distance [10]. However, in the case in which , the triangular inequality can be disrupted.

A solution to this problem is to apply a correction *a posteriori*, by summing to the distance a surcharge that depends on the number of unique markers. The triangular inequality holds for the function *m^id^*(*X*, *Y*) = *d^DCJ^*^–^*^id^* (*X*, *Y*) + *k · u*(*X*, *Y*), taking any constant *k* ≥ 3/2. Recall that *u*(*X*, *Y*) is the number of unique markers between genomes *X* and *Y.* We then have  and . Observe that *m^id^* depends only on the DCJ-indel distance and the number of unique markers.

The lower bound of 3/2 for the constant *k* was obtained by an overestimation for the DCJ-indel *diameter*, that is the maximum DCJ-indel distance between any two genomes *A* and *B.* It was also conjectured in [10] that the lower bound for the constant *k* could be reduced to 1.

## Results and discussion

The main results of this paper are a framework to assign weights to different operations in a hybrid model and the use of this framework to establish the triangular inequality for both the DCJ-indel and the DCJ-substitution distances.

### Framework to assign weights in a hybrid model

Let *w*(*ρ*) be the weight of an operation *ρ.* We propose a framework in which we have *w*(*ρ*) = 1 if *ρ* is an organizational operation. For each content-modifying operation *ρ*, we denote by *m*(*ρ*) the number of markers affected by *ρ*, that is the number of inserted or deleted markers. In the case of a substitution *ρ*, *m*(*ρ*) counts the markers that are deleted plus the markers that are inserted by *ρ.* The weight of *ρ* is then defined as *w*(*ρ*) = *p* + *km*(*ρ*), that is a linear function on the number *m*(*ρ*), with non-negative constants *p* and *k.* This framework adds *a priori* a surcharge *km*(*ρ*) to any content-modifying operation *ρ*.

Consider a generic hybrid model *H* that assigns to the rearrangement operations the weights given by the framework described above. Observe that, in a sequence of operations sorting a genome *A* into a genome *B* under *H*, each unique marker is affected by only one content-modifying operation:

**Proposition 1*** Given two genomes A and B*, *free of duplicated markers*, *and a generic hybrid model H*, *if**are the content-modifying operations in a sequence sorting A into B under H*, *then*.

We denote by  the distance between genomes *A* and *B* under *H.* We will first show in the following that for any positive *k*, the distance  is equivalent to the distance  up to an *a posteriori* correction made to the second distance.

**Lemma 3*** Given two genomes A and B without duplicated markers*, *where u*(*A*,*B*) *is the number of unique markers between A and B.*

*Proof:* Recall that *w*(*ρ*) = 1 if *ρ* is organizational, and *w*(*ρ*) = *p* + *km*(*ρ*) if *ρ* is content-modifying and affects *m*(*ρ*) markers. Consider a parsimonious sequence of operations *s* corresponding to the distance . Denote by  the organizational operations and by  the content-modifying operations in *s.* Observe that . Since *u*(*A*, *B*) is constant for a pair of genomes *A* and *B*,  is determined by choosing a sequence *s* that minimizes the value *r* + *tp* – such a value is exactly the distance .

From an algorithmic point of view, the relation established by Lemma 3 means that, when using this framework, one may focus on distances of type  – and the distance  can be easily obtained with the application of a simple *a posteriori* correction. In other words, the advantage of applying a correction *a posteriori* is that it does not interfere with the formula to compute the distance obtained without considering the correction.

We can derive from the previous observations a simpler inequality that can be used to determine the constant *k*:

**Proposition 2 ***Given three genomes A*, *B and C without duplicated markers*, *the inequality**holds if*, *and only if*, , *where**is the set of markers common only to A and B.*

*Proof:* Consider the disjoint sets from Figure 3. The inequality  is equivalent to the inequality

which can be re-written as . Since  does not affect  and increases  and , we can always assume .

Consider without loss of generality that  and . In order to establish the triangular inequality for , we need to find a non-negative *k* such that the inequality given by Proposition 2 holds. We can analyze first the case in which we have . In this case, the triangular inequality holds for , as we can obtain from a generalization of a proposition proved in [10]:

**Proposition 3*** Given p* > 0 *and three genomes A*, *B and C without duplicated markers*, *such that A and B have no common marker that does not occur in C*, *and*, *then*.

*Proof:* Recall the disjoint sets from Figure 3. We know that  and, w.l.o.g., we also assume that . Let *s*_1_ be an optimal sequence sorting *A* into *C.* The sequence *s*_1_ has some content-modifying operations involving elements from  and  and some organizational operations. In the same way, an optimal sequence *s*_2_ sorting *C* into *B* has some content-modifying operations involving elements from  and  and also some organizational operations. Note that *s*_1_*s*_2_ is a valid sequence sorting *A* into *B* (no content-modifying operation is applied to common markers). Thus , otherwise there would be a valid sequence with weight smaller than  sorting *A* into *B*, which is a contradiction. Since  and , we have 

Observe that, if the inequality holds for , it holds for  for *k* ≥ 0. More generally:

**Lemma 4*** Given a positive constant p and a non-negative constant k*, *if the triangular inequality holds for*, *then the triangular inequality holds for*, *for any k*′ ≥ *k.*

The minimum value of *k* to guarantee the triangular inequality depends on the value of *p* and on the specific model that we consider. In the following we will determine the minimum *k* for the DCJ-indel and the DCJ-substitution distances, considering *p* = 1.

### The triangular inequality in the DCJ-indel and DCJ-substitution distances

We can estimate the maximum values for both the DCJ-indel and DCJ-substitution distances with the help of Table 1, in which we give the DCJ-distance, number of runs and potentials per component of the adjacency graph. Remark that all values in this table depend only on the number of vertices in the respective component. Furthermore, Lemma 5 shows that the number of vertices in *AG*(*A*, *B*) depends on the number of common markers and chromosomes in genomes *A* and *B.*

**Table 1:** For each possible component *P* in an adjacency graph we give the number of vertices, the DCJ distance (that can be obtained in [4]) and the maximum values for Λ(*P*), *λ*(*P*) and *σ*(*P*).

|*P*|	*d^DCJ^*(*P*)	max Λ(P)	max λ(P)	max *σ*(*P*)
1	0	1	1	1
2	0	2	2	1
3	1	3	2	1
4	1	4	3	2
5	2	5	3	2
6	2	6	4	2
7 ⋮ |*P*|	3 ⋮ ⌊(|*P*| – 1)/2⌋	7 ⋮ |*P*|	4 ⋮ ⌈(|*P*| + 1)/2⌉	2 ⋮ ⌈(|*P*| + 1)/4⌉

**Lemma 5 ***The number of vertices in AG*(*A*, *B*) *is given by*

|*AG*(*A*, *B*)| = 2*n* + *L_A_* + *S_A_* + *L_B_* + *S_B_*,

*where n is the number of common markers of A and B*, *and L_A_*, *S_A_*, *L_B_ and S_B _are*, *respectively*, *the number of linear chromosomes and circular singletons in genomes A and B.*

*Proof:* Recall that, except for the circular singletons, each vertex in *AG*(*A*, *B*) is defined by a pair of symbols {*γ*_1_, *γ*_2_}, where each *γ_i_* is the head or the tail of a marker, or a telomere. The head *g^h^* of each common marker *g* appears in two vertices of *AG*(*A*, *B*) as well as the tail *g^t^* of *g* appears in two vertices of *AG*(*A*, *B*). Moreover, for each linear chromosome, two telomeres appear in vertices of *AG*(*A*, *B*). Hence, the total number of symbols due to chromosomes that are not circular singletons - i.e. linear chromosomes and chromosomes that contain common markers – is (4*n* + 2*L_A_* + 2*L_B_* )/2 = 2*n* + *L_A_* + *L_B_.* This added to the number *S_A_* + *S_B_* of circular singletons gives the final number of 2*n* + *L_A_* + *S_A_* + *L_B_* + *S_B_*.

We can now find the minimum *k* for the DCJ-indel and DCJ-substitution distances, considering *p* = 1.

#### The DCJ-indel distance

We first observe that . Furthermore, the *a posteriori* correction that we proposed in [10] is a particular case of the framework above: for any *k* ≥ 3/2, . The lower bound of 3/2 was obtained by overestimating the maximum DCJ-indel distance. In the present section we show that the DCJ-indel distance  satisfies the triangular inequality if and only if *k* ≥ 1. Such result solves an open conjecture mentioned in [10].

Lemma 6 determines a tight upper bound for the DCJ-indel distance between two genomes.

**Lemma 6 ***If A and B are genomes with n common markers*, *then*

*where L_A_*, *S_A_*, *L_B _and S_B _are*, *respectively*, *the number of linear chromosomes and circular singletons in genomes A and B.*

*Proof:* Recall from [10] that . Now, we study the maximum  per component, with the help of Table 1. If |*P*| is even, then *P* can be sorted with |*P*|/2 – 1 DCJs and at most *λ*(*P*) ≤ |*P*|/2 + 1 indels, which gives . If |*P*| is odd, then *P* can be sorted with (|*P*| – 1)/2 DCJs and at most *λ*(*P*) ≤ (|*P*| + 1)/2 indels, which gives . Summing  per component gives:

We can then reduce to 1 the lower-bound to the constant k, also proving that it is the best possible.

**Theorem 2*** The distance**satisfies the triangular inequality if and only if k* ≥ 1.

*Proof:* Let *A*, *B* and *C* be three genomes, with  and . Consider again the disjoint sets from Figure 3 and recall that, to prove the triangular inequality for , we only need to find a *k* such that  holds (Proposition 2). The case in which  is covered by Proposition 3. It remains to examine the case in which .

We need to characterize the worst configuration of genomes *A*, *B* and *C* so that we can find the smallest value for *k.* We know that genomes *A* and *B* are non-empty. Suppose that *C* is also non-empty (but remember that ). Observe that, in order to minimize *d^DCJ^*^–^*^id^*(*A*,*C*), the elements of  must be “together” in a single chromosome (in both genomes), not “intercalating” elements from  (the distance *d^DCJ^*^–^*^id^*(*A*,*B*) can be maximized “intercalating” only ). In this case, we can assume that the contibution of  in *d^DCJ^*^–^*^id^*(*A*, *C*) is zero, while the number of indels given by  in *d^DCJ^*^–^*^id^*(*B*, *C*) is equal to 1. We can then simply “move” all markers of , “removing” them from genome *C*, so that  , the number of indels between *A* and *B* is preserved, *d^DCJ^*^–^*^id^*(*A*, *C*) increases by 1 (one indel) and *d^DCJ^*^–^*^id^*(*B*,*C*) decreases by 1. Analogously, we can also consider that . With a similar analysis, we observe that the elements of  must be “together” in a single chromosome (in each of the three genomes), not “intercalating” elements from . Again, we can simply “move” all markers of , “removing” them from genome *C*, so that  and both *d^DCJ^*^–^*^id^*(*A*, *C*) + *d^DCJ^*^–^*^id^* (*B*, *C*) and *d^DCJ^*^–^*^id^*(*A*, *B*) are preserved. Thus, the worst case would be to have an empty genome *C.*

Let *X_A_*, *X_B_* be the number of chromosomes in *A* and *B*, *L_A_*, *L_B_* be the number of linear chromosomes in *A* and *B*, and *S_A_*, *S_B_* be the number of circular singletons in *A* and *B.* Since *C* is empty, we know that . From Lemma 6, we have  . This gives . Since *X_A_* ≥ *L_A_* + *S_A_* and *X_B_* ≥ *L_B_* + *S_B_*, we have , that holds for any *k* ≥ 1.

To show that the lower bound of 1 is tight, we take *k* = 1 – *ε*. Let *C* be the empty genome and let *A* and *B* be two unichromosomal circular genomes such that: (1) ; and (2) *AG*(*A*, *B*) has only one cycle, in which all vertices are labeled. Remark that  and . Moreover, . Thus, if *n* > 1/*ε*, we have *n*(1 – *k*) > 1 which is equivalent to 2*n* + 2*nk* > 2 + 4*nk* and also to , disrupting the inequality.

#### The DCJ-substitution distance

In the case of the DCJ-substitution distance, we also have . We find a lower bound to the constant *k* with the help of Lemma 7, that determines the maximum  between two genomes.

**Lemma 7*** If A and B are genomes with n common markers*, *then*

*where L_A_*, *S_A_*, *L_B_ and S_B_ are*, *respectively*, *the number of linear chromosomes and circular singletons in genomes A and B.*

*Proof:* Recall from [11] that . Now, we study  per component, with the help of Table 1, considering an integer *x* ≥ 0.

If |*P*| is even, then *P* can be DCJ-sorted with |*P*|/2 – 1 DCJs. We have to analyze two cases: (i) if |*P*| = 4*x* + 4, then *σ*(*P*) ≤ |*P*|/4 + 1 and ; (ii) if |*P*| = 4*x* + 2, then *σ*(*P*) ≤ (|*P*| – 2)/4 + 1 and . If |*P*| is odd, then *P* is an *AA-* or a *BB*-path and can be DCJ-sorted with (|*P*| – 1)/2 DCJs. Again, we have to analyze two cases: (i) if |*P*| = 4*x* + 3, then *σ*(*P*) ≤ (|*P*| + 1)/4 and ; (ii) if |*P*| = 4*x* + 1, then *σ*(*P*) ≤ (|*P*| + 3)/4 and . In this last case we could have *d^DCJ^*^–^*^sb^*(*P*) > 3|*P*|/4. Observe however that the numbers of *AA-* and *BB*-paths are bounded, respectively, by *L_A_* and *L_B_*. Summing  per component gives:

We can then establish a lower bound of 3/4 to the constant *k*, that is the best possible.

**Theorem 3 ***The distance**satisfies the triangular inequality if and only if k* ≥ 3/4.

*Proof:* The value of 3/4 is obtained by a procedure similar to the one in the proof of Theorem 2, except that here the maximum distance between two genomes is estimated as 3*n*/2 + *L_A_* + *L_B_* + *S_A_* + *S_B_* (Lemma 7). Supposing that *k* = (3 – *ε*)/4, we also show that the lower bound of 3/4 is tight.

### Discussion

Although the weights applied to content-modifying operations were motivated by the inequality disruption, we observe that they also lead to distances that are biologically more plausible. Consider again the example with genomes and the DCJ-indel distance. In this case we have the inequality disruption for  with  and . Using the ghost-DCJ model [12], that avoids the inequality disruption, the distances are *d*(*A*, B) = 3 and *d*(*A*, *C*) = *d*(*B*, *C*) = 2. Indeed, here the inequality holds, but these distances suggest that the phylogenetic relation between *A* and B is weaker than those between *A* and *C* or B and *C*, which would not be expected, since genomes *A* and *B* have the same content. We will now see what happens when we use , that gives  and . Observe that, with this correction, not only the inequality is established, but also the resulting distances suggest that the phylogenetic relation between *A* and *B* is stronger than those between *A* and *C* or *B* and *C.*

## Conclusions

When computing the distance between genomes with unequal content, the triangular inequality can be disrupted, so that the resulting distance does not constitute a metric. We show that we can correct this problem by selecting consistent weights for those genomic operations that change the content and those operations that change the organization of a genome. We describe a general framework for the correction of genomic distances that use both types of operations. Furthermore, we apply this framework to our DCJ-indel and DCJ-substitution distances, so that they satisfy the triangular inequality. This correction can be applied *a posteriori*, without interfering with the already available formulas to compute the distances under these models. We claim that this correction leads to distances that are biologically more plausible, regarding the phylogenetic relations between species.

### Future work

A natural extension of the present study is to apply the proposed framework to establish the triangular inequality in the inversion-indel distance.

Furthermore, the results of the present paper point to two clear avenues of research. The first one is to deeply investigate the distances  and  when *p* ≠ 1. In this case, it is not yet clear how to compute the distances and, consequently, it is not known which are the lowest values for *k* such that  and  satisfy the triangular inequality. The second avenue of research is to investigate weight functions different from *km*(*ρ*) + *p*, but this seems to be even more complicate. In fact, if the weight function is non-linear, even the correspondence between the *a priori* and *a posteriori* models is lost. In the near future, we also intend to evaluate the performance of the distances corrected by our framework in phylogenetic reconstructions.

## Competing interests

The authors declare that they have no competing interests.

## Authors’ contributions

MDVB, RM, LCR and JS have elaborated the model, proved the results and written the paper.
